# Do cancer stem cells exist? A pilot study combining a systematic review with the hierarchy-of-hypotheses approach

**DOI:** 10.1371/journal.pone.0225898

**Published:** 2019-12-13

**Authors:** Isabelle Bartram, Jonathan M. Jeschke

**Affiliations:** 1 Department of Biology, Chemistry, Pharmacy, Institute of Biology, Freie Universität Berlin, Berlin, Germany; 2 Leibniz-Institute of Freshwater Ecology and Inland Fisheries (IGB), Freie Universität Berlin, Berlin, Germany; 3 Berlin-Brandenburg Institute of Advanced Biodiversity Research (BBIB), Freie Universität Berlin, Berlin, Germany; Southern Illinois University School of Medicine, UNITED STATES

## Abstract

The phenomenon of cancer cell heterogeneity has been explained by different hypotheses, each entailing different therapy strategies. The most recent is the cancer stem cell model, which says that tumourigenicity and self-renewal are restricted to rare stem cell-like cancer cells. Since its conception, conflicting evidence has been published. In this study, we tested the applicability of a new approach developed in the field of ecology, the hierarchy-of-hypotheses approach, for the Cancer Stem Cell hypothesis. This approach allows to structure a broad concept into more specific sub-hypotheses, which in turn can be connected to available empirical studies. To generate a dataset with empirical studies, we conducted a systematic literature review in the Web of Science limited to the first 1000 publications returned by the search. From this pool, 51 publications were identified that tested whether a cell sub-population had cancer stem cell properties. By classifying the studies according to: (1) assessed indicators, (2) experimental assays and (3) model cancer cells used, we built a hierarchical structure of sub-hypotheses. The empirical tests from the selected studies were subsequently assigned to this hierarchy of hypotheses, and the percentage of supporting, undecided and questioning evidence was calculated for each sub-hypothesis, as well as additional experimental characteristics. Our approach successfully allowed us to determine that within our dataset, the empirical support for the CSC hypothesis was only 49.0%. The support of different sub-hypotheses was highly variable. Most noticeable, the conception that putative cancer stem cells are a rare subset of cells could not be confirmed by most studies (13.5% support). The empirical support varied also between types of cancer, animal models and cell isolation method used. For the first time, this study showed the applicability of the hierarchy-of-hypotheses approach for synthesizing and evaluating empirical evidence for a broad hypothesis in the field of bio-medical research.

## Introduction

The search for new cancer therapies is exacerbated by the fact that cancer is a highly heterogeneous disease. Cancer cells do not only vary phenotypically between patients and affected organs, but already within single tumours. This phenomenon was first explained by a clonal or stochastic model of cancer, where the heterogeneity of the cells is due to continuing mutagenesis [1,2,3]. Here, all cells have tumourigenic potential and, under the right external conditions, can re-establish a tumour. Early on it was challenged by the observation that in different kinds of cancers only a very small fraction of tumour cells proliferated when seeded in vitro or in vivo in mice [4,5] and that only certain primary tumour cells are capable to form metastasis in distant sites [6].

When Bonnet & Dick showed that in Acute Myeloid Leukaemia (AML), this subset of cells had an exclusive phenotype (CD34+/CD38–) [7], a new model to explain cancer cell heterogeneity was proposed: cancer stem cells (CSC) [8,9]. Here, a tumour is seen as an “abnormal organ” to which “the principles of normal stem cell biology can be applied” [8]. Analogous to normal tissues, the heterogeneous cell population of a tumour is exclusively replenished by multipotent CSCs that are able to self-renew and give rise to phenotypically different cells through asymmetric cell divisions. This concept is supported by the observation that many of the pathways that regulate self-renewal in stem cells were found to be active in cancer cells, such as the Wnt, the Sonic hedgehog (Shh) and the Notch pathway [8,10]. It also offers an explanation to the riddle why some cancer patients stay, seemingly cancer free, in remission for prolonged episodes of time before they relapse. Quiescence, a property of stem cells, would give CSCs the ability to survive treatment with chemotherapy in a dormant state to later re-enter the cell cycle and repopulate a tumour cell population [11].

While similar evidence was found for many cancer types, the CSC model has since been challenged as well. For each of the unique characteristics that were used to define CSCs as a separate population of cells (rarity, specific surface markers, tumourigenicity, differentiation potential, unlimited capacity for self-renewal, resistance against chemotherapy), contradictory evidence was found [12]. Additionally, further research has indicated that xenotransplantation, the “gold standard for in vivo testing” of the CSC model [11], might not be sufficient proof for an exclusive tumourigenicity of cells identified as CSCs. Quitana et al. found a surprising 25% of melanoma cells to be capable of tumourigenesis when they were injected into even more immunocompromised NOD/SCID IL2Rγnull mice (NGS) [13,14]. These tumourigenic cells could not be defined by CD133 or any other previously identified CSC marker and were phenotypically highly heterogeneous. It is thus arguable whether previous studies simply selected for cells with a unique capability to proliferate in mice rather than tumourigenic potential in humans. Another explanation for the conflicting results could be that some forms of cancer are indeed hierarchically organized with CSCs at the top, while others, like melanoma, are not.

Furthermore, a combination of both models is possible where CSCs do exist and are clonally selected, explaining the co-existence of different phenotypes of CSCs in studies on AML and glioblastoma [15,16]. Or maybe CSCs do exist but rather describe a transient state of cancer cells, which are able to switch their phenotype [17,18].

The two theoretical models are highly relevant because they result in different therapeutic strategies [19]. In the stochastic model, drugs must have a cytotoxic effect on all the heterogeneous cancer cell types of a tumour. In contrast, the existence of CSCs would have the attractive implication that it is only necessary to target a small fraction of cells in order to eradicate cancer from a patient. Additionally, stem cells have mechanisms of chemotherapy resistance such as a high expression of ABC transporters that if shared with CSCs need to be addressed in therapy development [9]. Accordingly, several new therapeutic strategies have been developed in the last years based on the assumption that CSCs are the drivers of cancer pathogenesis and progression. A focal area of research on these therapies has been the development of monoclonal antibodies or vaccines against the identified CSC markers such as CD133 or CD44, and small molecule inhibitors of receptors expressed predominantly by CSCs [20,21,22], although some researchers have criticized this strategy [12,19,23,24].

### The hierarchy-of-hypotheses approach (HoH)

While much basic research exists on CSC, a comprehensive synthesis and re-evaluation of the available evidence is currently lacking. Thus, even while CSC-specific therapies are already being developed and clinically tested [25], it remains contested whether CSC exist or not. As with most topics in biomedical basic research, the existing reviews on CSC are traditional narrative reviews that do not specify the process by which the authors have selected the included studies. On the other hand, formal meta-analysis *sensu stricto* can only be applied to research results that are either given in the same effect size (e.g. Hedges’ g) or are reasonably transformable to the same effect size [26]. Even if it is mathematically possible to express results in the same effect-size metric, it is not always clear if this is advisable, as effect sizes and their interpretation can sometimes genuinely vary among model systems, methods or spatiotemporal scales. To address these challenges of research synthesis, a method was recently developed within the field of ecology. This method is called the hierarchy-of-hypotheses (HoH) approach. One of its purposes is to help systematically analyse and contextualize empirical data and information. Thus far, the approach has been applied to test popular concepts in the field of invasion ecology for their actual empirical support [27,28,29,30]. The approach can be combined with formal meta-analytical methods.

In the HoH approach, the overarching hypothesis is divided into sub-hypotheses to create a hierarchical structure into which every empirical study in question is then sorted. By assessing the amount of support for each sub-hypothesis on the lowest hierarchical level, the overall support for higher-level sub-hypotheses and the overarching hypothesis can be assessed. The HoH approach allows to reduce complex hypotheses and makes it possible to visualize the network of relationships between (sub-) hypotheses, thereby also pointing out current research gaps [31]. Thus, an HoH analysis of existing experimental data on CSCs will make it possible to gain conceptual clarity and systematically test empirical support for this important and influential hypothesis on a large scale.

This study is a first feasibility test of the HoH approach in the biomedical research field, specifically for basic CSC research. Its objective is to investigate if the HoH approach is a useful method to structure the highly variable existing evidence surrounding the CSC model, and if it is helpful to test its different sub-hypotheses, e.g. the individual characteristics that are used to define CSC. Applying the HoH approach, it should also be possible to assess the amount of support for the CSC model in different research contexts such as type of cancer, used model organism or cell lines.

## Materials & methods

To generate a dataset with all available empirical studies on cancer heterogeneity, we conducted a systematic literature review following the PRISMA-P 2015 guidelines for systematic reviews and meta-analyses (see PRISMA diagram in S1 Fig; PRISMA checklist in S1 Table) [32]. First, we systematically searched the Web of Science (WoS) that tested the broad definition of the CSC hypothesis. Using the terms “cancer stem cells” or “tumor-initiating cells” or “tumour-initiating cells” or “tumorigenic cells” or “tumourigenic cells” or “tumor heterogeneity” or “tumour heterogeneity" on 6 September 2016. As this work is a first test whether the HoH approach is a useful method for CSC research synthesis in principle, we limited the scope of the study to the first 1000 publications (sorted by publication date) out of a total of 14,291 publications returned by the search. The truly relevant publications were identified by their titles and abstracts (returning 115 possibly relevant publications) and then thoroughly reading the publications in question. In this way, we identified a sample of 51 publications that tested whether a certain cell sub-population had CSC properties–independently of whether they were called as such or not. We thereby relied on the consensus definition of stem cells from the AARC cancer stem cell workshop [32], which defines a CSC as “a cell within a tumour that possess the capacity to self-renew and to cause the heterogeneous lineages of cancer cells that comprise the tumour.” Additional characteristics ascribed to CSCs when the concept was conceived and still implemented by current studies are rarity and the expression of stem cell markers [8,33]. We thus selected all studies that tested whether a subset of cancer cells complied with these characteristics compared to bulk tumour cells. Studies that only used cells the authors defined as CSCs and did not compare them to the rest of the cancer cell population were rejected. We also excluded theoretical (modelling) studies and reviews. In other words, the HoH analysis only included primary empirical studies.

From each of these 51 publications, we extracted the separate empirical tests the authors had performed, and recorded for each of them in a database: cancer type, model cancer cells (primary tumour cells, cell line, patient-derived xenograft …), animal model, CSC isolation method, indicator, assay, number of patient samples or cell lines tested, and result of the respective test.

To build an HoH of the CSC model, we defined different sub-hypotheses by classifying the evidence on three levels: (1) indicators assessed to test the existence of CSCs, (2) experimental assay and (3) model cancer cells used. On the 1st level of sub-hypotheses, the different indicators that are used to define CSCs in contrast to the bulk of cancer cells are located. As outlined above, these are the indicators ubiquitously used as defining characteristics of CSCs [8,33,34]. Further down, the 2nd level of sub-hypotheses contains the type of assays used to measure the respective indicators. For instance, differences in tumourigenicity between certain cancer cell subsets can be assessed by different empirical tests: by comparing the overall tumourigenic capacity of cell subsets (as measured by respective latency, number of tumours induced or overall tumour volume) after subcutaneous injections or orthotopic transplantations of cancer cells in vivo; or by comparing the frequency of tumour-inducing cells determined by limiting dilution injections in vivo. On the 3rd level of sub-hypotheses, the different origins of cells used in the assays are discriminated: human or other species, cultured or primary cancer tissue samples.

Finally, each empirical test recorded in the systematic review above was assigned to the sub-hypotheses at the lowest level, and was classified as either supporting, being undecided or questioning the sub-hypothesis in question (if the evidence was conflicting because e.g. the results from different cell lines tested were contradictory, the test was classified as “undecided”). For this scoring approach, we considered primarily effect sizes and the results of statistical significance tests given by the respective publications. The following specifications were made: the characteristic “rarity” is a major aspect of the CSC concept, but is not statistically evaluated in most studies. Here, a percentage of cells under 1% of the whole cell population was defined as “rare”. Another specification was necessary for assessing the support from expression analysis of RNA or protein markers. In many papers, several markers are assessed and therefore we set a cut-off at more than 50% of measured genes or proteins that had to be significantly differentially expressed to be counted as “supporting” evidence. If no measured stem-cell marker was differentially expressed, the evidence was counted as “questioning”, in-between as “undecided”. Many tumourigenicity assays only included a very small number of mice, and study authors often did not calculate statistical significance. But the evidence was still counted as “supporting” under the condition that tumours did form when the cell set defined as CSC was injected, while no tumours formed in mice when bulk tumour cells were injected. Please note that the HoH approach can also be combined with a fully quantitative meta-analytic framework if effect sizes are comparable, but this was not the case here, thus we applied the semi-quantitative scoring approach outlined in this paragraph.

To evaluate the support for the CSC model, the percentage of supporting, undecided and questioning evidence was calculated for each sub-hypothesis on all levels of the HoH. To account for the fact that most studies included several empirical tests, which are thus not independent of each other, each publication was only included once for each sub-hypothesis or the main hypothesis. Additionally, the amount of support was calculated in the same manner for different types of cancer, different model organisms and types of cancer cells used. We used a Mann-Whitney U-test to test for significant differences between the empirical support for sub-hypotheses and between different kinds of cancer etc. where the number of studies was ≥5. Statistical tests were performed using the software PAST version 3.23 (March 2019) [35].

## Results

**Fig 1:**
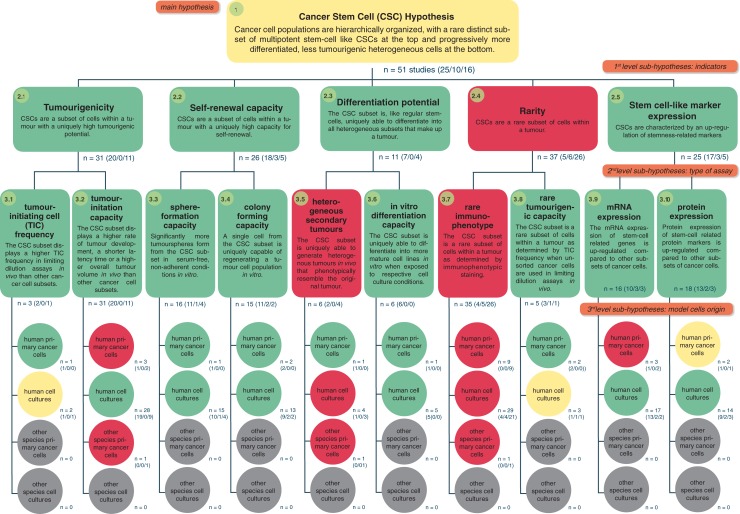
Hierarchy-of-hypotheses for the Cancer Stem Cell (CSC) hypothesis. The sub-hypotheses were defined by classifying the evidence on three levels: (1) indicators assessed to test the existence of CSCs, (2) experimental assay and (3) model cancer cells used. The empirical tests of analysed 51 studies were sorted into the HoH. Green indicates that more than 50% of the studies supported the sub-hypothesis; red indicates that more than 50% questioned the sub-hypothesis; yellow indicates sub-hypotheses for which neither supporting nor questioning evidence reached 50%. The number of studies with supporting / undecided / questioning evidence is indicated for each hypothesis in parentheses.

The 51 relevant publications that we identified studied putative CSCs in very different kinds of cancer using different model systems and different laboratory methods. As the authors tested several components of the CSC hypothesis in each study, the resulting number of empirical tests was 174 (on average 3–4 tests per study). A table with all empirical tests analysed is included in the S2 Table. We then assigned these tests to the respective sub-hypotheses and evaluated the empirical support for each of them and, due to the HoH approach, also for broader, higher-level sub-hypotheses. Fig 1 outlines the HoH structure for the CSC hypothesis, including the number of empirical tests summarized on each level and the amount of support for each sub-hypothesis.

**Fig 2:**
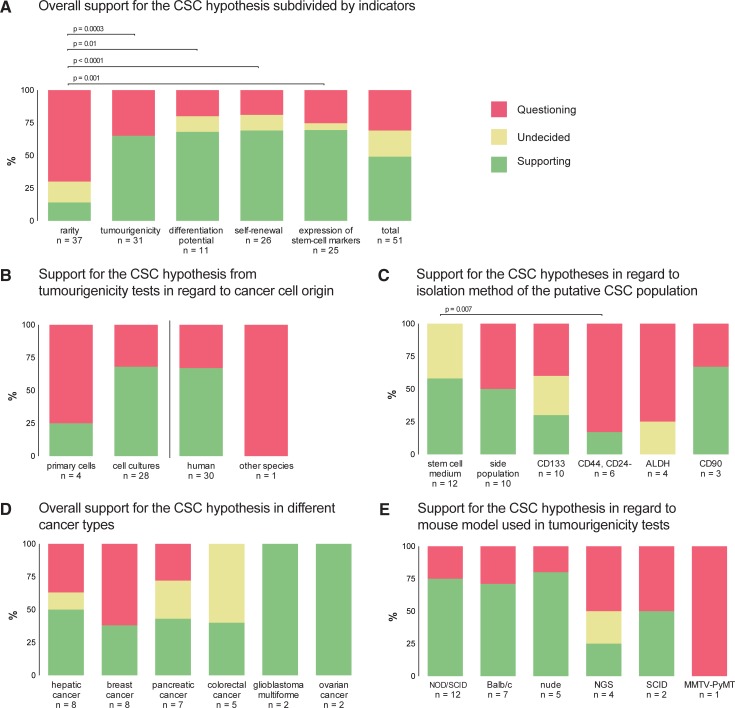
Empirical evidence regarding sub-hypotheses and further aspects of the Cancer Stem Cell hypothesis. The empirical evidence of each evaluated test was categorized as either supporting (S), being undecided (U) or questioning (Q), based on the data reported in the respective studies. N is the numbers of studies that tested the sub-hypotheses. Where the level of empirical support was significantly different (p<0.05, Mann Whitney U-tests), the respective p-value is specified.

The detailed results of the HoH analysis are illustrated in Fig 2. Within the 51 studies, the overall empirical support was 49.0% and highly variable for each second-level sub-hypothesis (Fig 2A). Most noticeable, the conception that putative CSCs are a rare subset of cells (for this analysis defined as 1% of tumour cells or less) could not be confirmed by most studies (13.5% support). The amount of supporting evidence was significantly higher in all other sub-hypotheses on the second level of the HoH.

The analysis also indicates that the support for the CSC concept varies between what cells were used–while most experiments using primary tumour cells did not support the CSC model, those that used cultured cells mostly confirmed it (Fig 2B). While more than half of the studies that used cells that grew selectively in stem-cell medium or expressed CD90 as putative CSCs found supporting evidence, studies that used other surface markers or side population staining did not. The level of support for the CSC model also varied greatly by which cancer type was investigated. While all studies from research on glioblastoma multiforme and ovarian cancer found evidence for the existence of CSCs, most studies in our dataset that investigated breast cancer did not (Fig 2D). Likewise, the support varied with the mouse model used in the studies. While most tumourigenicity tests using the NOD/SCID mouse model could confirm a CSC subset (75.0%), the level of support from tests using the more immunodeficient NGS mouse model was low (25.0%, Fig 2E).

## Discussion

Applying the HoH approach to studies in CSC research revealed highly interesting results, but of course a drawback of this pilot study is that only a fraction of the available literature was analysed. Extrapolating from this analysis, another 700–800 relevant publications are available in the literature.

Our results demonstrate that a comprehensive evaluation of all CSC studies available would be very valuable for the research community. For example, the notion that CSCs are a rare subset of cells within a tumour could face challenge if most tests reject this hypothesis also in a larger dataset. This would have implications for the idea that it is possible to develop drugs targeting a small number of CSCs to eradicate cancer from a patient. Also, several of the markers like CD133, which was thought by some to be a universal CSC marker [22], failed to define CSCs in a significant portion of empirical tests. Possibly the CSC phenotype is defined by different markers in different cancer types after all. A future lager dataset should also record cancer sub-types, as it is possible that even sub-types could feature different CSC markers.

In our analysis, the majority of tumourigenicity assays did show that the ability to form tumours was indeed limited to the subset of putative CSCs. But as others have observed [14], outcomes varied greatly by the used mouse model. This finding was not associated with different experimental contexts, e.g. different cancer types. Thus, the mouse model itself potentially influences the experimental outcome. The often used NOD-SCID mice are highly immunocompromised, but still tumour tissues often fail to engraft as some immune function remains. NGS mice additionally bear a mutation in the interleukin-2 gamma chain receptor that renders them highly receptive to engraftment of human cells [36]. Our results indicate that tumourigenicity assays used in the evaluated studies could simply select for cells that are good at evading the immune system of immunocompromised mice, not cells uniquely able to generate new tumours. Furthermore, experiments that used primary human cells from patients more often failed to prove the existence of CSCs in tumourigenicity tests. In contrast, the majority of experiments using cultured cells supported the CSC model. Replicated in a larger dataset, this could challenge the notion that cultured cells can be used as a simple substitute for gaining knowledge on tumours in patients. This is of course not a new observation–a recent study showed that the breast cancer cell line MCF7, which was also used in five studies in our dataset, is very different from the original patient sample and has become highly unreliable due to its many genetic and epigenetic alterations gained in culture [37].

If these results can be confirmed in a larger analysis, the implications for CSC research would be immense. One explanation for our findings could be that CSC is an unstable tumour cell state. Although when the CSC model was conceived it was (and still is) unclear how CSC emerge, the original model does not suggest that CSC is a highly plastic phenotype. Since then, some authors have proposed a redefinition where tumour cells can shift back and forth between CSC and non-CSC phenotype in response to environmental conditions as their findings did not match the model [17,18]. In our HoH, we applied the original hypothesis to first synthesise all available evidence and settle whether this original CSC model holds true–or which sub-hypotheses of the model are supported by evidence, and under which experimental conditions the CSC model can be corroborated. In a second step, a new model could then be proposed and tested. This approach would provide the urgently needed clarity of terminology, as the term CSC at this point has different meanings to different scientists.

In the field of invasion ecology, the HoH approach has helped to demonstrate that several intuitively appealing and widely used hypotheses are actually not supported by the majority of available empirical studies [30]. Similarly, this form of analysis could provide a useful tool to let go of disproven, but appealing hypotheses in the biomedical field–or provide a substantial empirical basis for their support. Additionally, the HoH analysis proved to be a useful tool to structure and categorize the seemingly heterogeneous research results surrounding the concept of CSCs. Up close, the 51 studies analysed all included very similar experiments to test the same characteristics. While it would have been difficult to summarize the unstructured results, the HoH analysis made it possible to compare different contexts and evaluate the support for the overarching hypotheses of the CSC model.

Basic research has recently been under much discussion for a possible “reproducibility crisis” with one culprit being the lack of published “negative” data, meaning results that do not show statistical differences or pointing in the “wrong” direction [38,39,40,41]. There has been a special concern for published basic cancer research after two pharmaceutical companies revealed that they could only reproduce a fraction of landmark studies [42]. In our preliminary analysis, we noticed that a major part of publications used different cell lines for different assays without giving an explanation. A possible reason for this phenomenon is that they performed the assays with all cell lines, but only presented the data that demonstrated a significant difference. Of course, we do not know if that was really the case. Such a practice would have a highly distorting effect on the available evidence that would be mirrored even in the most comprehensive HoH analysis. Thus, an ideal HoH would also include unpublished results. A solution could be an interactive open-access online platform where researchers can enter their data and in turn receive detailed information on overall support for the different sub-hypotheses in the exact context they are investigating or plan to investigate.

A further challenge that is not unique to the HoH approach, but shared by all quantitative and semi-quantitative meta-analyses *sensu lato* is the question of interdependence. Some publications include multiple tests of different sub-hypotheses, which are obviously not independent of each other. Other authors publish such tests of different sub-hypotheses in several smaller publications, which are again not independent of each other. Frequently, the same data are re-used in different publications, sometimes by different author teams. Studies based on different data but done by the same people are also not independent of each other. Even studies from different research groups are not necessarily independent of each other, as scientists influence each other through educational, institutional and specialization ties [43]. Co-author network analyses from different scientific disciplines revealed that relationships between researchers are highly clustered in “small worlds” where knowledge and resources are shared [44]. While knowledge flows between scientists have long been a study subject within social and data sciences by means of network analysis [43,45,46], to our knowledge these insights have not yet been integrated into meta-analyses on biomedical (or any other natural science) research. A first effort in this direction is a recent study from the field of biogeography [47] in which there was indeed a statistical association between the group of authors conducting a study and the outcome of the study. In further developing the HoH approach, these and other factors could be included in an “independence weight” of study outcomes, e.g. effect sizes reported in a study.

## Supporting information

S1 Fig PRISMA 2009 flow diagram. From Moher D, Liberati A, Tetzlaff J, Altman DG, The PRISMA Group (2009). Preferred reporting items for systematic reviews and meta-analyses: The PRISMA Statement. PLoS Med 6(6): e1000097. doi:10.1371/journal.pmed1000097. For more information, visit http://www.prisma-statement.org.
(TIF)

S1 Table PRISMA 2009 checklist. From: Moher D, Liberati A, Tetzlaff J, Altman DG, The PRISMA Group (2009). Preferred reporting items for systematic reviews and meta-analyses: The PRISMA statement. PLoS Med 6(7): e1000097. doi:10.1371/journal.pmed1000097
(DOC)

S2 Table Dataset of empirical tests. Tests and results from 51 studies testing the Cancer Stem Cell hypothesis that were included in our analysis.
(PDF)
