# Supplementary figures and images for: Do cancer stem cells exist? A pilot study combining a systematic review with the hierarchy-of-hypotheses approach
Source: PLoS One. 2019 Dec 13;14(12):e0225898. doi: 10.1371/journal.pone.0225898 (PMC6910685; doi:10.1371/journal.pone.0225898)

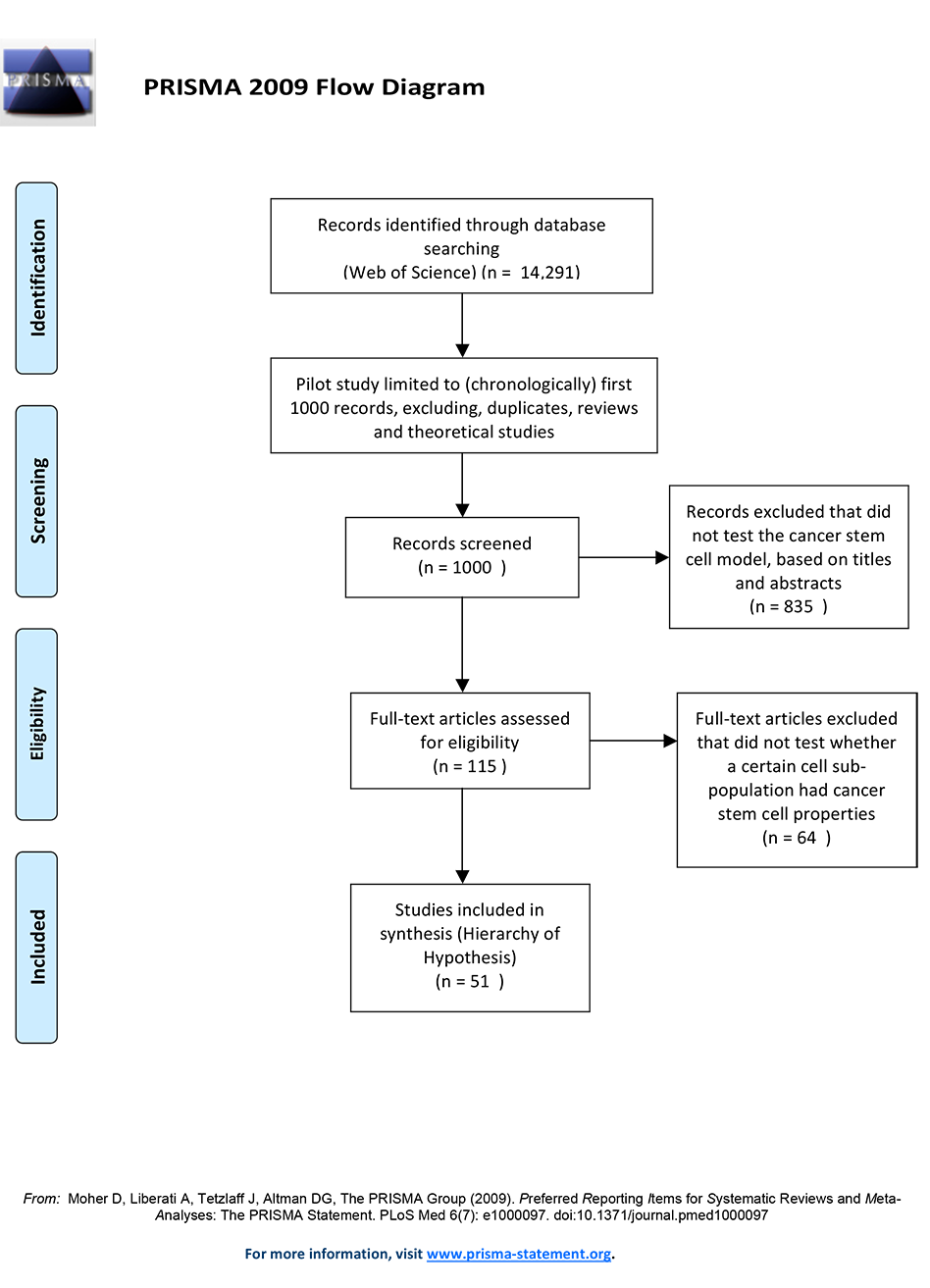

Supplement: S1 Fig — From Moher D, Liberati A, Tetzlaff J, Altman DG, The PRISMA Group (2009). Preferred reporting items for systematic reviews and meta-analyses: The PRISMA Statement. PLoS Med 6(6): e1000097. doi:10.1371/journal.pmed1000097. For more information, visit http://www.prisma-statement.org. (TIF) [file pone.0225898.s001.tif]
