# Supplementary material for: Do cancer stem cells exist? A pilot study combining a systematic review with the hierarchy-of-hypotheses approach
Source: PLoS One. 2019 Dec 13;14(12):e0225898. doi: 10.1371/journal.pone.0225898 (PMC6910685; doi:10.1371/journal.pone.0225898)
Supplement: S2 Table — Tests and results from 51 studies testing the Cancer Stem Cell hypothesis that were included in our analysis. (PDF) [file pone.0225898.s003.pdf]

**S3 Table. Dataset of Empirical Tests.** Tests and results from 51 studies testing the Cancer Stem Cell hypothesis that were included in the analysis.

**Classification**

| Indicator | | Assay | |
|---|---|---|---|
| 2.1 | Tumourigenicity | 3.1 | Tumour-initiating cell (TIC) frequency |
| | | 3.2 | Tumour-initiation capacity |
| 2.2 | Self-renewal | 3.3 | Sphere formation capacity |
| | | 3.4 | Colony forming capacity |
| 2.3 | Differentiation potential | 3.5 | Heterogeneous secondary tumours |
| | | 3.6 | In vitro differentiation capacity |
| 2.4 | Rarity | 3.7 | Rare immunophenotype |
| | | 3.8 | Rare tumourigenic capacity |
| 2.5 | Stem cell-like marker expression | 3.9 | mRNA expression |
| | | 3.10 | Protein expression |

**Abbreviations**

**Cell models**

| | |
|---|---|
| MDCL | mouse derived cell line |
| MDSC | mouse derived sphere culture |
| MDX | mouse derived xenograft |
| PDCL | patient derived cell line |
| PDX | patient derived xenograft |
| CLDX | cell line derived xenograft |
| CDSC | cell line sphere culture |
| PTS | primary tumour samples |

**Cancer type**

| | |
|---|---|
| ACC | adenoid cystic carcinoma |
| ATC | anaplastic thyroid cancer |
| CRC | colorectal cancer |
| ESCC | esophageal squamous cell carcinoma |
| GBM | glioblastoma multiforme |
| GC | gastric cancer |
| HCC | heptocellular carcinoma |
| HNSCC | head and neck squamous cell carcinoma |
| HR-NB | high-risk neuroblastoma |
| NPC | nasopharyngeal carcinoma |
| NSCLC | non-small cell lung carcinoma |
| OC | ovarian cancer |
| OSCC | oral squamous cell carcinoma |
| PanNET | pancreatic neuroendocrine tumors |
| PDAC | pancreatic ductal adenocarcinoma |
| RCC | renal cell carcinoma |

| Study ID | Pub. date (online) | DOI | Cancer Type | Model cells | Species | Model organism | CSC isolation method | Indicator | Assay | n (model cells) | Tests / study | Result |
|---|---|---|---|---|---|---|---|---|---|---|---|---|
| | | | | | | a.t. = athymic, nude | | | | n.s. = not specified | | S = supporting<br>Q = questioning<br>U = undecided |
| 1 | 23/12/2014 | 10.1007/s10616-014-9806-0 | CRC | CLDX | human | | selective growth in stem-cell medium | 2.3 | 3.6 | 1 | 4 | S |
| 1 | 23/12/2014 | 10.1007/s10616-014-9806-0 | CRC | CLDX | human | | selective growth in stem-cell medium | 2.5 | 3.10 | 1 | 4 | S |
| 1 | 23/12/2014 | 10.1007/s10616-014-9806-0 | CRC | CLDX | human | mouse, SCID | selective growth in stem-cell medium | 2.1 | 3.2 | 1 | 4 | Q |
| 1 | 23/12/2014 | 10.1007/s10616-014-9806-0 | CRC | CLDX | human | | selective growth in stem-cell medium | 2.5 | 3.9 | 1 | 4 | S |
| 2 | 16/01/2016 | PMID; 26862521 | GC | cell line | human | | selective growth in stem-cell medium | 2.5 | 3.9 | 1 | 2 | U |
| 2 | 16/01/2016 | PMID; 26862521 | GC | PTS | human | | selective growth in stem-cell medium | 2.5 | 3.9 | 1 | 2 | Q |
| 3 | 08/06/2016 | 10.3892/ol.2016.4690 | GBM | cell line | human | | retention of fluorescent membrane marker (Dil) | 2.3 | 3.6 | 3 | 5 | S |
| 3 | 08/06/2016 | 10.3892/ol.2016.4690 | GBM | cell line | human | | retention of fluorescent membrane marker (Dil) | 2.5 | 3.10 | 3 | 5 | Q |
| 3 | 08/06/2016 | 10.3892/ol.2016.4690 | GBM | cell line | human | mouse, NOD/SCID | retention of fluorescent membrane marker (Dil) | 2.1 | 3.2 | 3 | 5 | S |
| 3 | 08/06/2016 | 10.3892/ol.2016.4690 | GBM | cell line | human | | retention of fluorescent membrane marker (Dil) | 2.2 | 3.3 | 3 | 5 | S |
| 3 | 08/06/2016 | 10.3892/ol.2016.4690 | GBM | cell line | human | | retention of fluorescent membrane marker (Dil) | 2.4 | 3.7 | 3 | 5 | Q |
| 4 | 13/05/2016 | 10.3892/ol.2016.4565 | OC | PDCL | human | mouse, NOD/SCID | SP: Hoechst 33342 | 2.1 | 3.2 | 1 | 5 | S |
| 4 | 13/05/2016 | 10.3892/ol.2016.4565 | OC | PDCL | human | mouse, NOD/SCID | SP: Hoechst 33342 | 2.1 | 3.2 | 1 | 5 | S |
| 4 | 13/05/2016 | 10.3892/ol.2016.4565 | OC | PDCL | human | | SP: Hoechst 33342 | 2.2 | 3.4 | 1 | 5 | S |
| 4 | 13/05/2016 | 10.3892/ol.2016.4565 | OC | PDCL | human | | SP: Hoechst 33342 | 2.4 | 3.7 | 1 | 5 | S |
| 4 | 13/05/2016 | 10.3892/ol.2016.4565 | OC | PDCL | human | | SP: Hoechst 33342 | 2.2 | 3.3 | 1 | 5 | S |
| 5 | 20/06/2016 | 10.1158/0008-5472.CAN-15-2377 | melanoma | PDX | human | mouse, NGS | CD271 | 2.1 | 3.1 | 2 | 7 | Q |
| 5 | 20/06/2016 | 10.1158/0008-5472.CAN-15-2377 | melanoma | PDX | human | mouse, NGS | CD271 | 2.1 | 3.2 | 2 | 7 | Q |
| 5 | 20/06/2016 | 10.1158/0008-5472.CAN-15-2377 | melanoma | PTS | human | mouse, NGS | CD271 | 2.1 | 3.2 | 7 | 7 | Q |
| 5 | 20/06/2016 | 10.1158/0008-5472.CAN-15-2377 | melanoma | PDX | human | mouse, NGS | CD271 | 2.3 | 3.5 | 2 | 7 | Q |
| 5 | 20/06/2016 | 10.1158/0008-5472.CAN-15-2377 | melanoma | PDX | human | | CD271 | 2.4 | 3.7 | 6 | 7 | Q |
| 5 | 20/06/2016 | 10.1158/0008-5472.CAN-15-2377 | melanoma | PTS | human | | CD271 | 2.4 | 3.7 | 12 | 7 | Q |
| 5 | 20/06/2016 | 10.1158/0008-5472.CAN-15-2377 | melanoma | PDX | human | mouse, NGS | CD271-/+ | 2.4 | 3.8 | 2 | 7 | Q |
| 6 | 27/04/2016 | 10.3892/etm.2016.3303 | osteosarcoma | cell line | human | | SP: Hoechst 33342 | 2.5 | 3.10 | 1 | 4 | S |
| 6 | 27/04/2016 | 10.3892/etm.2016.3303 | osteosarcoma | cell line | human | | SP: Hoechst 33342 | 2.2 | 3.3 | 1 | 4 | S |
| 6 | 27/04/2016 | 10.3892/etm.2016.3303 | osteosarcoma | cell line | human | | SP: Hoechst 33342 | 2.4 | 3.7 | 1 | 4 | Q |
| 6 | 27/04/2016 | 10.3892/etm.2016.3303 | osteosarcoma | cell line | human | | SP: Hoechst 33342 | 2.5 | 3.9 | 1 | 4 | S |
| 7 | 23/03/2016 | 10.1016/j.canlet.2016.03.034 | OC | cell line | human | mouse, nude | CD133 | 2.1 | 3.2 | 1 | 3 | Q |
| 7 | 23/03/2016 | 10.1016/j.canlet.2016.03.034 | OC | cell line | human | mouse, nude | CD133 | 2.3 | 3.5 | 1 | 3 | S |
| 7 | 23/03/2016 | 10.1016/j.canlet.2016.03.034 | OC | cell line | human | | CD133 | 2.3 | 3.6 | 1 | 3 | S |
| 8 | 29/03/2016 | 10.1016/j.canlet.2016.03.041 | RCC | cell line | human | | SP: Hoechst 33342 | 2.4 | 3.7 | 1 | 1 | Q |
| 9 | 29/05/2016 | 10.18632/oncotarget.9654 | pancreatic cancer | PDX | human | mouse, NOD/SCID | unsorted | 2.4 | 3.8 | 11 | 1 | U |
| 10 | 13/04/2016 | 10.1158/0008-5472.CAN-15-2946 | breast cancer | PTS | mouse | mouse, MMTV-PyM | CD29hi, CD61, ALDH- | 2.1 | 3.2 | 1 | 4 | Q |
| 10 | 13/04/2016 | 10.1158/0008-5472.CAN-15-2946 | breast cancer | PTS | mouse | mouse, MMTV-PyM | ALDH | 2.1 | 3.2 | 1 | 4 | Q |
| 10 | 13/04/2016 | 10.1158/0008-5472.CAN-15-2946 | breast cancer | PTS | mouse | mouse, MMTV-PyM | CD29hi, CD61, ALDH- | 2.3 | 3.5 | 1 | 4 | Q |
| 10 | 13/04/2016 | 10.1158/0008-5472.CAN-15-2946 | breast cancer | PTS | mouse | mouse, MMTV-PyM | ALDH | 2.3 | 3.5 | 1 | 4 | Q |
| 11 | 06/04/2016 | 10.1089/scd.2016.0032 | insulinoma | cell line | human | mouse, a.t. | CD90 | 2.1 | 3.2 | 1 | 3 | S |
| 11 | 06/04/2016 | 10.1089/scd.2016.0032 | insulinoma | cell line | human | | CD90 | 2.4 | 3.7 | 1 | 3 | Q |
| 11 | 06/04/2016 | 10.1089/scd.2016.0032 | pancreatic cancer | cell line | human | | CD90 | 2.4 | 3.7 | 1 | 3 | Q |
| 12 | 04/09/2015 | 10.3109/1061186X.2015.1082567 | breast cancer | cell line | human | | CD44, CD24− | 2.2 | 3.3 | 1 | 3 | S |
| 12 | 04/09/2015 | 10.3109/1061186X.2015.1082567 | breast cancer | cell line | human | | CD44, CD24− | 2.4 | 3.7 | 3 | 3 | U |
| 12 | 04/09/2015 | 10.3109/1061186X.2015.1082567 | breast cancer | cell line | human | | CD44, CD24− | 2.5 | 3.9 | 1 | 3 | S |
| 13 | 26/05/2016 | 10.1186/s12885-016-2372-4 | breast cancer | cell line | human | | ALDH+ | 2.2 | 3.3 | 5 | 4 | U |
| 13 | 26/05/2016 | 10.1186/s12885-016-2372-4 | breast cancer | cell line | human | | SP: Hoechst 33342 | 2.4 | 3.7 | 5 | 4 | Q |
| 13 | 26/05/2016 | 10.1186/s12885-016-2372-4 | breast cancer | cell line | human | | CD44, CD24–/low | 2.4 | 3.7 | 5 | 4 | Q |
| 13 | 26/05/2016 | 10.1186/s12885-016-2372-4 | breast cancer | cell line | human | | ALDH+ | 2.4 | 3.7 | 5 | 4 | Q |

| | | | | | | | | | | | | |
|---|---|---|---|---|---|---|---|---|---|---|---|---|
| 14 | 09/04/2016 | 10.18632/oncotarget.8665 | ACC | cell line | human | mouse, nude | CD133 | 2.1 | 3.2 | 1 | 2 | S |
| 14 | 09/04/2016 | 10.18632/oncotarget.8665 | ACC | cell line | human | | CD133 | 2.3 | 3.6 | 1 | 2 | S |
| 15 | 18/04/2016 | 10.18632/oncotarget.8830 | liver cancer | cell line | human | mouse, NOD/SCID | selective growth in stem-cell medium | 2.1 | 3.2 | 1 | 3 | S |
| 15 | 18/04/2016 | 10.18632/oncotarget.8830 | liver cancer | cell line | human | | selective growth in stem-cell medium | 2.5 | 3.9 | 1 | 3 | S |
| 15 | 18/04/2016 | 10.18632/oncotarget.8830 | liver cancer | cell line | human | | selective growth in stem-cell medium | 2.2 | 3.4 | 1 | 3 | Q |
| 16 | 17/05/2016 | 10.1186/s12943-016-0521-7 | esophageal cancer | cell line | human | | selective growth in stem-cell medium | 2.5 | 3.10 | 2 | 4 | Q |
| 16 | 17/05/2016 | 10.1186/s12943-016-0521-7 | esophageal cancer | cell line | human | mouse, Balb/c | selective growth in stem-cell medium | 2.1 | 3.2 | 1 | 4 | S |
| 16 | 17/05/2016 | 10.1186/s12943-016-0521-7 | esophageal cancer | cell line | human | | selective growth in stem-cell medium | 2.5 | 3.9 | 2 | 4 | U |
| 16 | 17/05/2016 | 10.1186/s12943-016-0521-7 | esophageal cancer | cell line | human | | selective growth in stem-cell medium | 2.2 | 3.4 | 1 | 4 | S |
| 17 | 30/03/2016 | 10.1073/pnas.1604721113 | GBM | CLDX | human | mouse, NGS | CD133, GD3 | 2.1 | 3.1 | 1 | 7 | S |
| 17 | 30/03/2016 | 10.1073/pnas.1604721113 | GBM | cell line | human | | selective growth in stem-cell medium | 2.5 | 3.10 | 4 | 7 | S |
| 17 | 30/03/2016 | 10.1073/pnas.1604721113 | GBM | cell line | human | mouse, NGS | selective growth in stem-cell medium | 2.1 | 3.2 | 1 | 7 | Q |
| 17 | 30/03/2016 | 10.1073/pnas.1604721113 | GBM | cell line | human | | selective growth in stem-cell medium | 2.2 | 3.3 | 1 | 7 | Q |
| 17 | 30/03/2016 | 10.1073/pnas.1604721113 | GBM | CLDX | human | | CD133, GD3 | 2.2 | 3.3 | 1 | 7 | S |
| 17 | 30/03/2016 | 10.1073/pnas.1604721113 | GBM | CLDX | human | | CD133, GD3 | 2.2 | 3.3 | 1 | 7 | S |
| 17 | 30/03/2016 | 10.1073/pnas.1604721113 | GBM | cell line | human | | selective growth in stem-cell medium | 2.5 | 3.9 | 4 | 7 | S |
| 18 | 21/03/2016 | 10.1074/jbc.M115.700021 | HNSCC | cell line | human | mouse, NOD/SCID | CD44hi, ALDH1hi | 2.1 | 3.2 | 1 | 2 | S |
| 18 | 21/03/2016 | 10.1074/jbc.M115.700021 | HNSCC | cell line | human | | CD44hi, ALDH1hi | 2.4 | 3.7 | 1 | 2 | Q |
| 19 | 12/02/2016 | 10.1038/gt.2016.15 | CRC | cell line | human | mouse, Balb/c | selective growth in stem-cell medium | 2.1 | 3.2 | 3 | 2 | Q |
| 19 | 12/02/2016 | 10.1038/gt.2016.15 | CRC | cell line | human | | selective growth in stem-cell medium | 2.2 | 3.4 | 3 | 2 | S |
| 20 | 17/03/2016 | 10.3892/ol.2016.4343 | HCC | cell line | human | mouse, nude | SP: Hoechst 33342 | 2.1 | 3.2 | 1 | 4 | S |
| 20 | 17/03/2016 | 10.3892/ol.2016.4343 | HCC | cell line | human | | SP: Hoechst 33342 | 2.2 | 3.4 | 1 | 4 | S |
| 20 | 17/03/2016 | 10.3892/ol.2016.4343 | HCC | cell line | human | | SP: Hoechst 33342 | 2.4 | 3.7 | 4 | 4 | Q |
| 20 | 17/03/2016 | 10.3892/ol.2016.4343 | HCC | cell line | human | | SP: Hoechst 33342 | 2.5 | 3.9 | 1 | 4 | S |
| 21 | 05/04/2016 | 10.1016/j.biopha.2016.03.024 | breast cancer | cell line | human | | CD44, CD24− | 2.4 | 3.7 | 2 | 1 | Q |
| 22 | 10/03/2016 | 10.3892/ijo.2016.3432 | ESCC | cell line | human | mouse, Balb/c | p75NTR | 2.1 | 3.2 | 1 | 5 | S |
| 22 | 10/03/2016 | 10.3892/ijo.2016.3432 | ESCC | cell line | human | mouse, NOD/SCID | p75NTR | 2.1 | 3.2 | 1 | 5 | S |
| 22 | 10/03/2016 | 10.3892/ijo.2016.3432 | ESCC | cell line | human | | p75NTR | 2.2 | 3.4 | 2 | 5 | U |
| 22 | 10/03/2016 | 10.3892/ijo.2016.3432 | ESCC | cell line | human | | p75NTR | 2.4 | 3.7 | 2 | 5 | Q |
| 22 | 10/03/2016 | 10.3892/ijo.2016.3432 | ESCC | cell line | human | | p75NTR | 2.5 | 3.9 | 2 | 5 | S |
| 23 | 28/04/2016 | 10.1038/srep25220 | RCC | PTS | human | mouse, NGS | unsorted | 2.4 | 3.8 | 30 | 1 | S |
| 24 | 01/03/2016 | 10.18632/oncotarget.7803 | HCC | PTS | human | | GEPhi | 2.3 | 3.6 | n.s. | 6 | S |
| 24 | 01/03/2016 | 10.18632/oncotarget.7803 | HCC | PTS | human | | GEPhi | 2.5 | 3.10 | 42 | 6 | S |
| 24 | 01/03/2016 | 10.18632/oncotarget.7803 | HCC | PTS | human | mouse, NOD/SCID | GEPhi | 2.1 | 3.2 | 11 | 6 | Q |
| 24 | 01/03/2016 | 10.18632/oncotarget.7803 | HCC | PTS | human | | GEPhi | 2.2 | 3.3 | 3 | 6 | S |
| 24 | 01/03/2016 | 10.18632/oncotarget.7803 | HCC | PTS | human | | GEPhi | 2.2 | 3.4 | 6 | 6 | S |
| 24 | 01/03/2016 | 10.18632/oncotarget.7803 | HCC | PTS | human | | GEPhi | 2.4 | 3.7 | 42 | 6 | Q |
| 25 | 31/03/2016 | 10.1073/pnas.1600007113 | PanNET | PTS | human | mouse, NGS | CD90hi | 2.1 | 3.1 | 1 | 5 | S |
| 25 | 31/03/2016 | 10.1073/pnas.1600007113 | PanNET | PTS | human | mouse, NGS | CD90hi | 2.1 | 3.2 | 1 | 5 | S |
| 25 | 31/03/2016 | 10.1073/pnas.1600007113 | PanNET | PTS | human | mouse, NGS | CD90hi | 2.3 | 3.5 | 1 | 5 | S |
| 25 | 31/03/2016 | 10.1073/pnas.1600007113 | PanNET | PTS | human | | CD90hi | 2.4 | 3.7 | 1 | 5 | Q |
| 25 | 31/03/2016 | 10.1073/pnas.1600007113 | PanNET | PTS | human | mouse, NGS | unsorted | 2.4 | 3.8 | 1 | 5 | S |
| 26 | 14/04/2016 | 10.1158/1078-0432.CCR-15-2208 | ACC | PDX | human | mouse, nu/nu | CD133 | 2.1 | 3.2 | 1 | 4 | S |
| 26 | 14/04/2016 | 10.1158/1078-0432.CCR-15-2208 | ACC | PDX | human | mouse, nu/nu | CD133 | 2.3 | 3.5 | 1 | 4 | Q |
| 26 | 14/04/2016 | 10.1158/1078-0432.CCR-15-2208 | ACC | PDX | human | | CD133 | 2.4 | 3.7 | 5 | 4 | Q |
| 26 | 14/04/2016 | 10.1158/1078-0432.CCR-15-2208 | ACC | PDX | human | | CD133 | 2.5 | 3.9 | 5 | 4 | Q |
| 27 | 07/03/2016 | 10.18632/oncotarget.7954 | HCC | cell line | human | | CD133, ALDH1 | 2.2 | 3.3 | 1 | 2 | S |
| 27 | 07/03/2016 | 10.18632/oncotarget.7954 | HCC | cell line | human | | CD133, ALDH1 | 2.4 | 3.7 | 1 | 2 | Q |
| 28 | 06/04/2016 | 10.12659/MSM.895645 | laryngeal carcinoma | cell line | human | mouse, BALB/c-nu/ | CD133 | 2.1 | 3.2 | 1 | 3 | Q |
| 28 | 06/04/2016 | 10.12659/MSM.895645 | laryngeal carcinoma | cell line | human | | CD133 | 2.2 | 3.4 | 1 | 3 | Q |
| 28 | 06/04/2016 | 10.12659/MSM.895645 | laryngeal carcinoma | cell line | human | | CD133 | 2.4 | 3.7 | 1 | 3 | Q |

| | | | | | | | | | | | | |
|---|---|---|---|---|---|---|---|---|---|---|---|---|
| 29 | 24/12/2014 | 10.1002/hed.23975 | OSCC | cell line | human | | SP: Hoechst 33346 | 2.5 | 3.10 | 2 | 5 | S |
| 29 | 24/12/2014 | 10.1002/hed.23975 | OSCC | cell line | human | mouse, BALB/c nuc | SP: Hoechst 33345 | 2.1 | 3.2 | 1 | 5 | S |
| 29 | 24/12/2014 | 10.1002/hed.23975 | OSCC | cell line | human | | SP: Hoechst 33343 | 2.2 | 3.3 | 2 | 5 | S |
| 29 | 24/12/2014 | 10.1002/hed.23975 | OSCC | cell line | human | | SP: Hoechst 33344 | 2.2 | 3.4 | 2 | 5 | S |
| 29 | 24/12/2014 | 10.1002/hed.23975 | OSCC | cell line | human | | SP: Hoechst 33342 | 2.4 | 3.7 | 6 | 5 | Q |
| 30 | 06/11/2015 | 10.1007/s13277-015-4226-0 | breast cancer | cell line | human | | CD44, CD24− | 2.2 | 3.3 | 2 | 3 | Q |
| 30 | 06/11/2015 | 10.1007/s13277-015-4226-0 | breast cancer | cell line | human | | CD44, CD24− | 2.4 | 3.7 | 2 | 3 | Q |
| 30 | 06/11/2015 | 10.1007/s13277-015-4226-0 | breast cancer | PTS | human | | CD44, CD24− | 2.4 | 3.7 | 25 | 3 | Q |
| 31 | 07/03/2016 | 10.3892/mmr.2016.4986 | HCC | PTS | human | | SP: Hoechst 33345 | 2.5 | 3.10 | n.s. | 4 | Q |
| 31 | 07/03/2016 | 10.3892/mmr.2016.4986 | HCC | PTS | human | | SP: Hoechst 33345 | 2.2 | 3.4 | n.s. | 4 | S |
| 31 | 07/03/2016 | 10.3892/mmr.2016.4986 | HCC | PTS | human | | SP: Hoechst 33345 | 2.4 | 3.7 | n.s. | 4 | Q |
| 31 | 07/03/2016 | 10.3892/mmr.2016.4986 | HCC | PTS | human | | SP: Hoechst 33345 | 2.5 | 3.9 | n.s. | 4 | Q |
| 32 | 30/03/2016 | 10.4174/astr.2016.90.4.183 | CRC | cell line | human | | CD133 | 2.2 | 3.3 | 1 | 2 | Q |
| 32 | 30/03/2016 | 10.4174/astr.2016.90.4.183 | CRC | cell line | human | | CD133 | 2.4 | 3.7 | 1 | 2 | S |
| 33 | 28/12/2015 | 10.1111/cas.12870 | breast cancer | cell line | human | | selective growth in stem-cell medium | 2.5 | 3.10 | 1 | 5 | S |
| 33 | 28/12/2015 | 10.1111/cas.12870 | breast cancer | cell line | human | | CD44, CD24− | 2.4 | 3.7 | 2 | 5 | Q |
| 33 | 28/12/2015 | 10.1111/cas.12870 | breast cancer | PTS | human | | CD44, CD24− | 2.4 | 3.7 | 1 | 5 | Q |
| 33 | 28/12/2015 | 10.1111/cas.12870 | breast cancer | cell line | human | | selective growth in stem-cell medium | 2.5 | 3.9 | 1 | 5 | S |
| 33 | 28/12/2015 | 10.1111/cas.12870 | breast cancer | PTS | human | | selective growth in stem-cell medium | 2.5 | 3.9 | 1 | 5 | S |
| 34 | 07/10/2015 | 10.14670/HH-11-676 | CRC | cell line | human | mouse, nude | CD133 | 2.1 | 3.2 | 1 | 2 | S |
| 34 | 07/10/2015 | 10.14670/HH-11-676 | CRC | PTS | human | | CD133 | 2.4 | 3.7 | 19 | 2 | Q |
| 35 | 17/11/2015 | 10.1016/j.jhep.2015.11.011 | HCC | PTS | human | | SP: Hoechst 33346 | 2.4 | 3.7 | 18 | 1 | Q |
| 36 | 28/01/2015 | 10.1002/mc.22279 | NPC | cell line | human | mouse, NOD/SCID | SP: Hoechst 33346 | 2.1 | 3.2 | 1 | 5 | S |
| 36 | 28/01/2015 | 10.1002/mc.22279 | NPC | cell line | human | | SP: Hoechst 33346 | 2.2 | 3.4 | 1 | 5 | S |
| 36 | 28/01/2015 | 10.1002/mc.22279 | NPC | cell line | human | | SP: Hoechst 33346 | 2.4 | 3.7 | 1 | 5 | U |
| 36 | 28/01/2015 | 10.1002/mc.22279 | NPC | cell line | human | | SP: Hoechst 33346 | 2.2 | 3.3 | 1 | 5 | S |
| 36 | 28/01/2015 | 10.1002/mc.22279 | NPC | cell line | human | | SP: Hoechst 33346 | 2.5 | 3.9 | 1 | 5 | S |
| 37 | 19/12/2015 | 10.18632/oncotarget.6672 | HCC | cell line | human | | CD90 | 2.3 | 3.6 | 1 | 4 | S |
| 37 | 19/12/2015 | 10.18632/oncotarget.6672 | HCC | cell line | human | mouse, NOD/SCID | CD90 | 2.1 | 3.2 | 2 | 4 | S |
| 37 | 19/12/2015 | 10.18632/oncotarget.6672 | HCC | cell line | human | | CD90 | 2.4 | 3.7 | 7 | 4 | Q |
| 37 | 19/12/2015 | 10.18632/oncotarget.6672 | HCC | cell line | human | | CD90 | 2.2 | 3.4 | 1 | 4 | S |
| 38 | 30/01/2016 | 10.18632/oncotarget.7078 | pancreatic cancer | cell line | human | | CD133 | 2.5 | 3.10 | 2 | 5 | S |
| 38 | 30/01/2016 | 10.18632/oncotarget.7078 | pancreatic cancer | cell line | human | mouse, SCID | CD133 | 2.1 | 3.2 | 2 | 5 | S |
| 38 | 30/01/2016 | 10.18632/oncotarget.7078 | pancreatic cancer | cell line | human | | CD133 | 2.2 | 3.3 | 1 | 5 | S |
| 38 | 30/01/2016 | 10.18632/oncotarget.7078 | pancreatic cancer | cell line | human | | CD133 | 2.4 | 3.7 | 1 | 5 | S |
| 38 | 30/01/2016 | 10.18632/oncotarget.7078 | pancreatic cancer | cell line | human | | CD133 | 2.5 | 3.9 | 2 | 5 | S |
| 39 | 20/02/2016 | 10.1186/s12943-016-0501-y | rhabdomyosarcoma | cell line | human | | selective growth in stem-cell medium | 2.5 | 3.10 | 1 | 1 | U |
| 40 | 18/02/2016 | 10.1186/s13287-016-0291-6 | HR-NB | cell line | human | | selective growth in stem-cell medium | 2.5 | 3.10 | 1 | 2 | S |
| 40 | 18/02/2016 | 10.1186/s13287-016-0291-6 | HR-NB | cell line | human | | selective growth in stem-cell medium | 2.5 | 3.9 | 1 | 2 | S |
| 41 | 30/01/2016 | 10.18632/oncotarget.7084 | CRC | cell line | human | mouse, Balb/c | CD133hi, CD44hi | 2.1 | 3.2 | 1 | 4 | S |
| 41 | 30/01/2016 | 10.18632/oncotarget.7084 | CRC | cell line | human | | CD133hi, CD44hi | 2.2 | 3.3 | 1 | 4 | Q |
| 41 | 30/01/2016 | 10.18632/oncotarget.7084 | CRC | cell line | human | | CD133hi, CD44hi | 2.4 | 3.7 | 6 | 4 | U |
| 41 | 30/01/2016 | 10.18632/oncotarget.7084 | CRC | cell line | human | | CD133hi, CD44hi | 2.5 | 3.9 | 1 | 4 | S |
| 42 | 12/02/2016 | 10.1186/s12967-016-0785-0 | HCC | PTS | rabbit | rabbit, New Zealanc | ALDHhi | 2.4 | 3.7 | n.s. | 2 | Q |
| 43 | 12/12/2015 | 10.18632/oncotarget.6570 | NSCLC | cell line | human | | CD133 | 2.4 | 3.7 | 3 | 2 | U |
| 43 | 12/12/2015 | 10.18632/oncotarget.6570 | NSCLC | cell line | human | | CD133 | 2.5 | 3.10 | 3 | 1 | Q |
| 44 | 09/02/2016 | 10.1371/journal.pone.0148807 | PDAC | cell line | human | mouse, NGS | SP: Hoechst 33346 | 2.1 | 3.2 | 1 | 6 | Q |
| 44 | 09/02/2016 | 10.1371/journal.pone.0148807 | PDAC | CDSC | human | mouse, NGS | SP: Hoechst 33346 | 2.1 | 3.2 | 1 | 6 | Q |
| 44 | 09/02/2016 | 10.1371/journal.pone.0148807 | PDAC | cell line | human | mouse, NGS | SP: Hoechst 33346 | 2.3 | 3.5 | 1 | 6 | Q |
| 44 | 09/02/2016 | 10.1371/journal.pone.0148807 | PDAC | CDSC | human | mouse, NGS | SP: Hoechst 33346 | 2.3 | 3.5 | 1 | 6 | Q |
| 44 | 09/02/2016 | 10.1371/journal.pone.0148807 | PDAC | cell line | human | | SP: Hoechst 33346 | 2.4 | 3.7 | 2 | 6 | Q |

| | | | | | | | | | | | |
|---|---|---|---|---|---|---|---|---|---|---|---|
| 44 | 09/02/2016 | 10.1371/journal.pone.0148807 | PDAC | CDSC | human | | SP: Hoechst 33346 | 2.4 | 3.7 | 1 | 6 | Q |
| 45 | 31/12/2015 | 10.18632/oncotarget.6805 | HCC | cell line | human | | selective growth in stem-cell medium | 2.5 | 3.10 | 1 | 5 | S |
| 45 | 31/12/2015 | 10.18632/oncotarget.6805 | HCC | cell line | human | | selective growth in stem-cell medium | 2.5 | 3.10 | 1 | 5 | Q |
| 45 | 31/12/2015 | 10.18632/oncotarget.6805 | HCC | cell line | human | mouse, NOD/SCID | selective growth in stem-cell medium | 2.1 | 3.2 | 2 | 5 | S |
| 45 | 31/12/2015 | 10.18632/oncotarget.6805 | HCC | cell line | human | | selective growth in stem-cell medium | 2.2 | 3.4 | 2 | 5 | S |
| 45 | 31/12/2015 | 10.18632/oncotarget.6805 | HCC | cell line | human | | selective growth in stem-cell medium | 2.5 | 3.9 | 1 | 5 | Q |
| 46 | 12/02/2016 | 10.1080/15384101.2015.1127471 | pancreatic cancer | cell line | human | | selective growth in stem-cell medium | 2.5 | 3.10 | 3 | 2 | S |
| 46 | 12/02/2016 | 10.1080/15384101.2015.1127471 | pancreatic cancer | cell line | human | mouse, NOD/SCID | selective growth in stem-cell medium | 2.1 | 3.2 | 1 | 2 | Q |
| 47 | 16/07/2015 | 10.1093/neuonc/nov123 | medulloblastoma | PDX | human | | CD133 | 2.4 | 3.7 | 4 | 2 | Q |
| 47 | 16/07/2015 | 10.1093/neuonc/nov123 | medulloblastoma | PDX | human | | CD15 | 2.4 | 3.7 | 4 | 2 | Q |
| 48 | 07/01/2016 | 10.1016/j.lfs.2015.12.057 | ATC | cell line | human | | CD133 | 2.4 | 3.7 | 2 | 2 | Q |
| 48 | 07/01/2016 | 10.1016/j.lfs.2015.12.057 | ATC | cell line | human | | CD133 | 2.5 | 3.9 | 2 | 2 | S |
| 49 | 17/11/2015 | 10.3892/or.2015.4434 | breast cancer | cell line | human | | selective growth in stem-cell medium | 2.5 | 3.10 | 1 | 2 | S |
| 49 | 17/11/2015 | 10.3892/or.2015.4434 | breast cancer | cell line | human | mouse, BALB/c a.t. | selective growth in stem-cell medium | 2.1 | 3.2 | 1 | 2 | S |
| 49 | 17/11/2015 | 10.3892/or.2015.4434 | breast cancer | cell line | human | | selective growth in stem-cell medium | 2.2 | 3.4 | 1 | 2 | S |
| 50 | 18/12/2015 | 10.3892/ijo.2015.3299 | PanNET | cell line | human | mouse, NOD/SCID | ALDHhi | 2.1 | 3.2 | 1 | 5 | S |
| 50 | 18/12/2015 | 10.3892/ijo.2015.3299 | PanNET | cell line | human | | ALDHhi | 2.2 | 3.3 | 2 | 5 | S |
| 50 | 18/12/2015 | 10.3892/ijo.2015.3299 | PanNET | PTS | human | | ALDHhi | 2.4 | 3.7 | 15 | 5 | Q |
| 50 | 18/12/2015 | 10.3892/ijo.2015.3299 | PanNET | cell line | human | | ALDHhi | 2.4 | 3.7 | 2 | 5 | Q |
| 50 | 18/12/2015 | 10.3892/ijo.2015.3299 | PanNET | cell line | human | mouse, NOD/SCID | unsorted | 2.4 | 3.8 | 1 | 5 | S |
| 51 | 17/12/2015 | 10.1073/pnas.1522602113 | breast cancer | cell line | human | mouse, NOD/SCID | CD44, CD24-/lo, SSEA-3 | 2.1 | 3.2 | 1 | 6 | Q |
| 51 | 17/12/2015 | 10.1073/pnas.1522602113 | breast cancer | cell line | human | mouse, NOD/SCID | ESAhi, PROCRhi, SSEA-3 | 2.1 | 3.2 | 1 | 6 | Q |
| 51 | 17/12/2015 | 10.1073/pnas.1522602113 | breast cancer | cell line | human | mouse, NOD/SCID | SSEA-3 | 2.1 | 3.2 | 1 | 6 | Q |
| 51 | 17/12/2015 | 10.1073/pnas.1522602113 | breast cancer | cell line | human | | CD44, CD24-/lo, SSEA-3 | 2.2 | 3.3 | 1 | 6 | Q |
| 51 | 17/12/2015 | 10.1073/pnas.1522602113 | breast cancer | cell line | human | | ESAhi, PROCRhi, SSEA-3 | 2.2 | 3.4 | 1 | 6 | S |
| 51 | 17/12/2015 | 10.1073/pnas.1522602113 | breast cancer | cell line | human | | SSEA-3 | 2.4 | 3.7 | 1 | 6 | S |
